# Human Pregnancy and Parturition Clinical Management

**DOI:** 10.1155/2010/915875

**Published:** 2011-04-28

**Authors:** Faustino R. Pérez-López, Sean C. Blackwell, Edmund F. Funai, Shi-Wen Jiang, Marc J. N. C. Keirse, Liliana S. Voto

**Affiliations:** ^1^Department of Obstetrics and Gynecology, Faculty of Medicine, University of Zaragoza and Hospital Clínico, 50009 Zaragoza, Spain; ^2^Division of Maternal Fetal Medicine, Department of Obstetrics, Gynecology and Reproductive Sciences, The University of Texas Health Science Center at Houston, TX 77030, USA; ^3^Section of Maternal Fetal Medicine, Department of Obstetrics, Gynecology and Reproductive Sciences, Yale University School of Medicine, New Haven, CT 06520, USA; ^4^Department of Biomedical Science, Mercer University School of Medicine, Savannah, GA 31404, USA; ^5^Department of Obstetrics, Gynaecology and Reproductive Medicine, Flinders Medical Centre, Flinders University, Bedford Park, SA 5042, Australia; ^6^Departamento de Obstetricia y Ginecologia, Hospital Juan A. Fernández, Universidad de Buenos Aires, Cerviño 3356, Capital Federal, Buenos Aires, Argentina

Pregnancy and parturition are major events in human life. The health care providers' mission of antenatal care is to treat conditions that may alter development and health of the embryo and fetus. In consequence, birth and parturition represent a challenge for the adaptation to a new environment. Birth conditions may vary in relation to healthcare facilities, prenatal care, and educational and economical conditions. A major challenge for those assisting human birth is to properly and timely identify mothers and fetuses at risk for complications. Globalization of information disseminated through mass media and the Internet has revealed the huge inequalities that women suffer during pregnancy and parturition. Healthiness among mothers and their children is a critical indicator of health status of the world's population as well as the health outcome of future generations.

Online scientific publications have increased during the last years as alternatives to conventional journals. Publications with the “online open access” modality are based on two aspects: (1) a peer-review system similar to that used for centuries in the Western World science and (2) publication cost is covered by the authors. Submission of a scientific contribution is generally performed seeking the highest impact possible. In order to compete with traditional journals, those online have to transverse a long journey in order to be evaluated by the scientific community and the appropriate organizations and to achieve an impact factor. These scenarios give rise to a Darwinian natural selection-like process in which final destination of a given contribution will depend on its intrinsic quality. Affecting this process are the autoevaluation of the authors and the rigor and editorial criteria of a given journal. Although not always true, higher impact journals receive contributions with higher quality whereas those with low or no impact factor the contrary.

For the occasion of this issue of *Obstetrics and Gynecology International,* 17 papers were submitted to the peer-review process. Publication criteria were not guided by any specific recommendation. It was based on the individual editors' expertise and experience and assessment of the reports received from independent referees. As a product of the review process, the readers will find 5 accepted contributions from different origin addressing several issues of human pregnancy and parturition management.

An international group of researchers (M. L. Kamb et al.) in their paper “*A Road Map for the Global Elimination of Congenital Syphilis*” review the problem of congenital syphilis and its impact over perinatal deaths. It was estimated that untreated syphilis in the world causes a similar or even higher mortality than HIV.

Researchers from The Netherlands (M. P. G. C. Vinken et al.) present in their paper “*Nifedipine-Induced Changes in the Electrohysterogram of Preterm Contractions: Feasibility in Clinical Practice*” a clinical study regarding the use of the electrohysterogram to monitor uterine contractions in women with preterm labor managed with nifedipine. 

Stillbirths and neonatal deaths are major issues in the care of pregnant women, especially in developing countries. Researchers affiliated to different Norwegian Institutions (A. Jammeh et al.) in their paper “*Jammeh et al. Stillbirths in Rural Hospitals in The Gambia: A Cross-Sectional Retrospective Study*” performed a cross-sectional retrospective study of stillbirth in rural Gambia. 

Authors from Ecuador and Spain (D. Salazar-Pousada et al.) in their paper “*Salazar-Pousada et al. Depressive Symptoms and Resilience among Pregnant Adolescents: A Case-Control Study*” present a comparative controlled study to determine resilience and depressive status among pregnant adolescents and compare them to young pregnant adults in their 20s. 

And finally, authors from the United States (A. W. Ayres and S. K. Pugh) in their paper “*Ayres and Pugh. Ex Utero Intrapartum Treatment for Fetal Oropharyngeal Cyst*” report on an oropharyngeal cyst compromising fetal airway which was managed with an ex utero intrapartum treatment procedure. Fetoplacental circulation was maintained until the fetal airway was secured.

*Faustino R. Pérez-López* *Faustino R. Pérez-López* *Sean C. Blackwell* *Sean C. Blackwell* *Edmund F. Funai* *Edmund F. Funai* *Shi-Wen Jiang* *Shi-Wen Jiang* *Marc J. N. C. Keirse* *Marc J. N. C. Keirse* *Liliana S. Voto* *Liliana S. Voto*

